# Behavior Variations and Their Implications for Popularity Promotions: From Elites to Mass on Weibo

**DOI:** 10.3390/e24050664

**Published:** 2022-05-09

**Authors:** Bowen Shi, Ke Xu, Jichang Zhao

**Affiliations:** 1 State Key Lab of Software Development Environment, Beihang University, Beijing 100191, China; bowenshi@buaa.edu.cn (B.S.); kexu@buaa.edu.cn (K.X.); 2 School of Economics and Management, Beihang University, Beijing 100191, China

**Keywords:** social media, user behavior, posting entropy, elites, user influence, popularity promotion

## Abstract

The boom in social media with regard to producing and consuming information simultaneously implies the crucial role of online user influence in determining content popularity. In particular, understanding behavior variations between the influential elites and the mass grassroots is an important issue in communication. However, how their behavior varies across user categories and content domains and how these differences influence content popularity are rarely addressed. From a novel view of seven content domains, a detailed picture of the behavior variations among five user groups, from the views of both the elites and mass, is drawn on Weibo, one of the most popular Twitter-like services in China. Interestingly, elites post more diverse content with video links, while the mass possess retweeters of higher loyalty. According to these variations, user-oriented actions for enhancing content popularity are discussed and testified. The most surprising finding is that the diverse content does not always bring more retweets, and the mass and elites should promote content popularity by increasing their retweeter counts and loyalty, respectively. For the first time, our results demonstrate the possibility of highly individualized strategies of popularity promotions in social media, instead of a universal principle.

## 1. Introduction

Content popularity is the key goal for advertisers, innovators and influentials in communication [1,2,3], and it heavily depends on the social influence and behaviors of its origins. For example, corporations have made efforts to find the right influentials for advertising, and organizers even employ zealots to influence voters [4,5]. With the flourishing of online social media, massive users are both producers and consumers, instead of only audiences [6], which thoroughly undermines the stereotypes of communication roles and essentially challenges the promotion of content popularity. However, the diversity of topical contexts in social media and multiple online behaviors and their implications for popularity promotions are rarely mentioned. In fact, only a small group of people have influence [3], but both ordinary people and elites want to become famous or more famous to create trends [7], which drives us to explore the impact of behavior and influence on popularity from the perspective of both the mass and influentials. It is worth noting that the comparison of behavioral differences between influentials and mass grassroots has always been one of the important issues in communication [7,8,9,10,11,12]. In addition, influentials are often referred to as “opinion leaders”, “innovators” or “early adopters” in the Diffusion of Innovation theory [3]. Given the ambiguity and lack of clarity of the phrase “opinion leader” [13], the term “elites” is employed to refer to influential users in this article, similar to many studies [14,15].

In recent decades, many scholars focused on the comparison of behavioral differences between elites and the mass [7,11,12,16], but they ignore some key elements. On the one hand, the behavior variations across various user groups and content domains were ignored in previous efforts and still remain unclear. While being new channels of information exchange, in addition to average citizens, social media also provide diverse conduits for users, such as news media, government agencies and enterprises [17]. Different user demographics might result in distinctive behaviors and influence [18,19]. For example, government officials actively interact with citizens regarding local issues [20] while enterprises focus on promoting products [21]; accordingly, their influence and popularity promotions should be treated differently. Moreover, Hilbert et al. summarized that communication contexts surely influence communication structures [22], and it is thus possible that various users may demonstrate different patterns in multiple domains. In particular, Usain Bolt definitely has a lot of fans in the sport-related domain on Twitter, while the mainstream media accounts, such as BBC, broadcast news on various aspects such as politics, society, sports and technology. Therefore, it is imperative that the behavioral differences between the mass and elites should be pictured across domains and user groups. On the other hand, the impact of behavioral differences between the mass and elites on their strategies to promote popularity has not been examined. Although a number of studies put forward some strategies to enhance content popularity [1,23,24,25,26], it is unclear whether these strategies are effective either for various user groups or various domains. For each elite and ordinary people who want to increase their influence, a mapping of strategy across user groups and domains is needed instead of simply imitating others. To fill the above void, we explore the impact of behavioral differences on the content popularity, helping the elites and mass with the right actions for popularity enhancement in different communication contexts.

To address the above gaps in the existing research, we determine to examine the following research questions (RQs):

- RQ1. What are the behavioral differences between the mass and elites across various user groups and content domains?
- RQ2. Are there differences between the actions of the mass and elites to promote content popularity?
- RQ3. How to choose promotion strategies suitable for various user groups and content domains?

As a result of the scarcity of massive user data in social sciences and the complexity of multiple domains, many traditional methods (e.g., questionnaires and surveys) are challenging to implement because of the spatial limitations and high costs [8,27]. Fortunately, the digital traces accumulated and aggregated in social media provide a more efficient but less expensive proxy for investigating the exact mapping between user groups and content domains [28,29,30]. More importantly, Weibo has attracted 500 million users in China, surpassing any other social networking sites in China [31], and extensive efforts have been devoted to study user behaviors on Weibo [7,12,23]. In particular, the authentication category system of Weibo provides an opportunity to further study the fine-grained user categories of the mass and elites. Meanwhile, determining the appropriate number of domains is a difficult task because of the complex contents on Weibo, so we use a topic classifier suitable for the Weibo discourse system based on machine learning to divide the domains. In addition, considering that the status of elites should be constantly developing and changing in interaction [32], users known as “Big Vs” but of no real influence will affect the results, and the traditional methods such as informants’ ratings and self-designation are subjective, biased and difficult to quantify the real influence of massive users [3,33]. On the contrary, we establish retweet networks to select elites that are really influential.

To investigate the comparison between the mass and elites across user groups and content domains in a data-driven manner, techniques and methods from machine learning and social network analysis are employed in this study. With the help of a topic classifier adapted to the discourse system on Weibo [34], we use the machine learning model to divide 140,000,000 tweets into seven main topic categories, such as society, sports and so on. Then, by collecting the retweets of 8.52 million users in seven domains, seven networks are established to identify the elites. We apply the position of a node in the topology to measure the importance of users [35] and ultimately selected 930 truly influential users. As for the category of user authentication, unlike Twitter, Weibo has a strict verified system which requires users to provide manual documentary evidence and divides them into five main categories such as media, government and so on. In particular, these verified users play crucial roles in the information dissemination on Weibo. Accordingly, the verified types can be a direct clue for grouping users. In fact, grouping all users into different clusters, on the one hand, will support the investigation of all participants in online communication instead of only elites and, on the other hand, will greatly reduce randomness at the individual level and make it feasible to stably map user behavior onto content domains at the collective level. In terms of splitting the contents into seven domains and apportioning users into these groups, the following investigation of how user behavior varies across content domains can be comprehensively conducted.

Tweeting and retweeting are the most frequent components of user behaviors on Weibo [36,37], and some scholars emphasized that content-specific attributes such as content links can also affect popularity [1,38]. On the basis of a well-established grouping system of users and domains, here the behavioral differences between the mass and elites are comprehensively probed from the perspectives of activity, homophily, loyalty and content characteristics. Based on the entropy characteristics [39], posting entropy is introduced to measure the diversity of content topics. We further attempt to figure out strategies fit for the behaviors of various users to specifically increase their content popularity. Through the comparison from multiple perspectives, many unexpected differences in behavior and strategy between the mass and elites are revealed. This study powerfully demonstrates that each user needs to choose the right ways to increase influence across domains, suggesting that the popularity promotion strategy is closely coupled with content domains and user groups. The exact mapping established here can directly help develop suitable strategies for popularity promotions in social media, which is particularly instrumental to market segmentation in target marketing [40]. Taking the action of adding links as an example, we demonstrate that the mapping between user groups and content domains can inspire ways to enhance popularity in a fine-grained manner, especially as both the user group and the content domain are the inputs of this practice. Additionally, the diverse perspectives are investigated, which further ensures the extendibility of our conclusions.

The main contributions of the paper are as follows:

- This study is the first to disclose the behavior variations from elites to the mass across user groups and multiple domains in social media. With regard to splitting users into five groups and the contents into seven domains, an accurate and complete spectrum of behavior variations across domains is comprehensively established. With the help of a spectrum, what kinds of users targeted as behaviorally influential seeds in marketing-like applications can be optimally pinpointed.
- Comprehensive mapping between behavior variations and popularity promotions is established in rich perspectives ranging from activity patterns to various content characteristics. In particular, though targeting influentials are extensively exploited, this is the first time to study the popularity promotion for the mass. Appropriate strategies for popularity enhancement can accordingly be derived from the mapping in terms of taking both user groups and content domains into account.
- Machine learning and network analysis are jointly employed in this study, which enriches the practical methodologies in probing massive users in a communication study. Driven by massive tweets and huge retweet networks on Weibo, solutions involving artificial intelligence and intensive calculations are conducted to split user groups, cut content domains and draw the mapping, overcoming the high costs and low efficiency of conventional approaches.

## 2. Literature Review

### 2.1. Differences in Behavior between Elites and the Mass

In social media, everyone is simultaneously a publisher and a listener of information, and all users equivalently constitute the communicator and audience elements in the communication model [6]. According to the two-step flow model [41], the propagation of information is a secondary dissemination process in which most people form their own views under the influence of elites, e.g., public opinion leaders. Opinion leaders, characterized as the influentials with more connections, are crucial for information dissemination [42,43]. In the meantime, the influentials hypothesis, in which influentials will trigger wide dissemination, has been questioned in recent years [7,44]. It has already been pointed out that the mass play a decisive role at the early stage of trend creation [7], implying that user influence can be counterintuitive and cannot be overly simplified, and elites and the mass have a gap in opinions [8,10]. Therefore, the comparison between elites and the public is a meaningful task that deserves more efforts.

As a key issue, many studies regarding the comparison between elites and the masses inherently neglected the behavior variations across user groups and content domains [7,11,12]. In particular, various user groups, e.g., professions, might result in different behaviors in online social media [18]. Enterprises advertise products [21] while athletic stars promote their popularity, and Zhao et al. [19] divided users into four categories (i.e., engineer, recruiter, salesperson and manager) that fit for Linkedin to study their behavioral differences. Moreover, Smith et al. [45] observed six different communication patterns in digital media, and contexts were also emphasized to feature different structures [22,46]. In understanding the behavior variation over multiple domains, it is also possible that elites may demonstrate patterns that differ from others. In this paper, to capture a complete picture, the behavior variation of elites across content domains and user groups is therefore separately discussed and compared with the case of all users, i.e., the mass level.

### 2.2. Behavior for Popularity Promotions

User behavior is a direct reflection of the information diffusion in which tweeting and retweeting are two primary activities on Weibo and have been exploited extensively in previous efforts. High activity, also known as the frequency of posting, indicates a greater likelihood of exposure [16,26,36]. Many users like to embed links of images, videos and news to make content charming in social media [47]. Moreover, retweeting is a crucial attribute in interactive behavior [37,48] and reflects the social homophily. The homophily refers to the fact that the individual prefers to have contact with people with many similar behavioral characteristics [49] and has been demonstrated in various social media [50,51,52,53]. Guillen et al. [54] also summarized that both customer loyalty and number growth had a positive impact on profits. Loyalty, another factor that impacts retweeting, is measured by the retweeting frequency and, in essence, reflects the multiple behavior properties such as interaction, satisfaction and intimacy. Nevertheless, the comparison between the behavior of the mass and elites is rarely performed on these different dimensions, implying the necessity for more comprehensive explorations.

Content popularity is the prime target in communication. Many factors underlying behaviors can affect content popularity, in particular, the narrative characteristics [1,3,55]. Intuitively, rich and diverse content will attract more audiences of different interests, but cognitive psychologists have long contended that human beings have a limited capacity for information processing [56]. Too rich topics can lead to a decrease in content quality in a single domain, thereby losing audiences and even popularity. It is also indicated that users on Weibo are quite keen on inserting short links jumping to news, pictures and videos into tweets [23,26,57]. In addition, loyal customers play an important role in maintaining a basic level of attention [58] and increasing loyalty can upgrade profits [59,60]. These factors could be potential features in popularity prediction and promotion. Szabo and Huberman used linear regression to predict the online popularity on YouTube and Digg [61], while Chen et al. [62] applied a binary classification model to identify the trend in a time series. However, they ignored the behavioral differences of various user clusters across content domains; after all, Figueiredo et al. [1] highlighted that the domain’s context is a crucial factor in changing popularity. Meanwhile, there is a lack of fine-grained recommendation on effective enhancement strategies. In this paper, content domains, user groups and other dimensions of behaviors such as loyalty and content diversity will be comprehensively integrated to target the right enhancement strategy for each situation.

## 3. Materials and Methods

### 3.1. Weibo Data Set

The Weibo data in this study were collected through its open API (application program interface). Over 140,000,000 tweets from the Weibo stream occurring from 10 October 2016 to 10 January 2017 were continuously crawled and, in total, we sampled 8,520,933 unique users. The signals delivered in these posts are sophisticated and are from every aspect of everyday life. Specifically, the JSON file of each tweet contains attributes of text, retweet status and user demographics such as the verified type, gender, address and the number of followers, suggesting that the content domains, user groupings and influence metrics can comprehensively be derived from these attributes. For each user, the tweeting frequency and retweet times are accumulatively counted based on the retweet status of the user’s tweets, and the other rarely updated demographics, such as gender and verified type, are obtained from the latest tweets in our data set.

### 3.2. User Groups

In particular, unlike Twitter, a distinctive verification mechanism on Weibo ensures the reliability of the user demographics, especially the verified types. On Weibo, users with certain verified types are known as the “Big Vs” [12] and the platform even demonstrates red or blue badges on their profiles. Specifically, in addition to the basic real-name certification for each ordinary user, further verification steps involve (1) a certain reputation and influence in specific domains, (2) well-known enterprises and their executives, (3) the mainstream media and (4) government agencies such as public authorities. Note that verification requires documentary evidence and is manually performed. More rigorously, enterprise users need to complete an Enterprise User Certification Information Form and Corporate Certification Application Letter and affix their corporate color seal and pay an annual fee. In general, the official verified types can be categorized in terms of the media, celebrity, government and enterprise. According to verified types, we can split the users into five groups, with the addition of those without verified types, i.e., ordinary users. Note that the authenticity of ordinary users can also be ensured due to the real-name certification regulation in China. The summary statistics of the user groups are in Table 1, with ordinary users accounting for the most and the government accounting for the least.

### 3.3. Domain Classifier

The main form of content on Weibo is text, and its topic can well represent the domain the content belongs to. Considering the massive text data, an automatic topic classifier is expected, and what is more, the appropriate number of domains is critical. In this study, a previously well-developed Naive Bayesian classifier is adopted to perform domain categorization [34]. The classifier is trained on more than 410,000 Weibo tweets and its seven topic categories fit well with the news taxonomy of Weibo. Based purely on text features, the domain classifier can divide a tweet into one of seven topics: society, international, sports, technology, entertainment, finance and military.

The model performance is shown in Appendix A Table A1. Both the F-measure and accuracy of the classifier in the cross-validation experiment is more than 84%, suggesting its sufficient competence in the domain classification task. Concretely, we can first convert the text of each tweet into a vector $w_{i}$, where $w_{i}$ and i refer to a term and its position in tweet t after the word segmentation. In the incremental training process, the prior probability of term $w_{i}$ belonging to topic c is calculated as

(1) $$P(w_{i}\left||c\right)=\frac{n^{c}\left(w_{i}+1\right)}{\sum_{q}n^{c}\left(w_{q}\right)+1},$$

where c belongs to the topic categories $C=(c_{1},c_{2},c_{3},c_{4},c_{5},c_{6},c_{7})$ and $n^{c}\left(w_{i}\right)$ indicates the count of occurrences of $w_{i}$ in topic c. Finally, the domain of a word vector is obtained by the maximum value of the probability calculated as $P\left(c|t\right)={\mathrm{argmax}}_{c}P\left(c\right)P(w_{i}\left||c\right)$, where P(c) is the prior probability of c. Note that tweets with ambiguous topics will be labeled “unknown” by the classifier. Before subsequent experiments, we filter out these topic-ambiguity tweets to eliminate the influence of topic relevance on our analysis. The average precision of this classifier is convincing and, in particular, the large number of tweets that we employ in the experiment can further guarantee its accuracy after the aggregation. Its mechanism of incremental training can also solve the problem of new words in to-do tasks. In terms of grouping users into five clusters by user groups, angles from both user groups and content domains can be thus established to investigate behavior variations.

### 3.4. Selection of Elites

**Figure 1:**
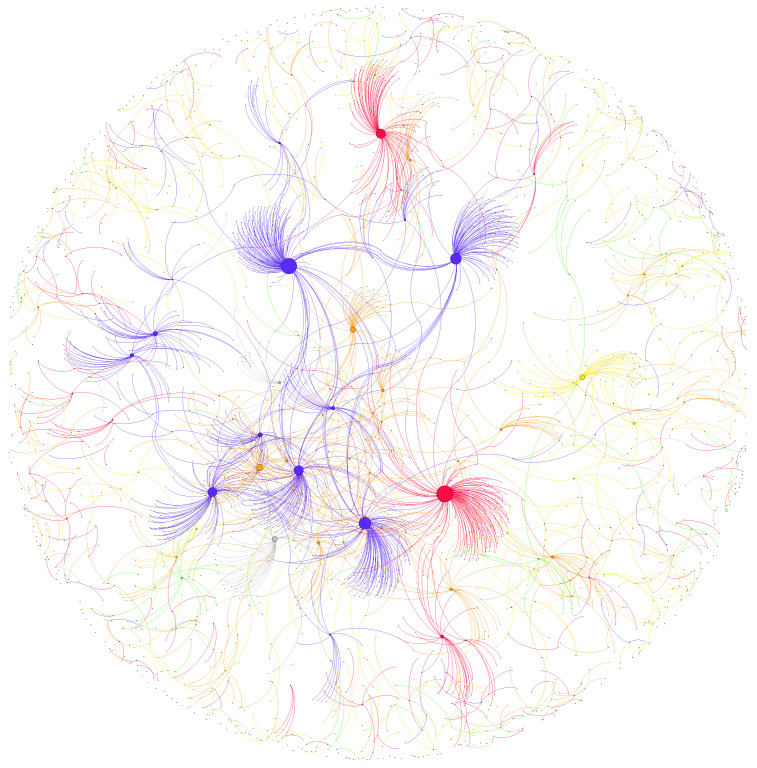
Retweet network of various users in the military domain. The threshold of the edge weight is set to 10, and the size of the node is related to its number of retweeters. We color each node by its verified type, i.e., blue represents the media, green represents enterprises, red represents the government, orange represents celebrities and gray represents others. Note that the color of the edge is the same as that of the source node.

**Figure A1:**
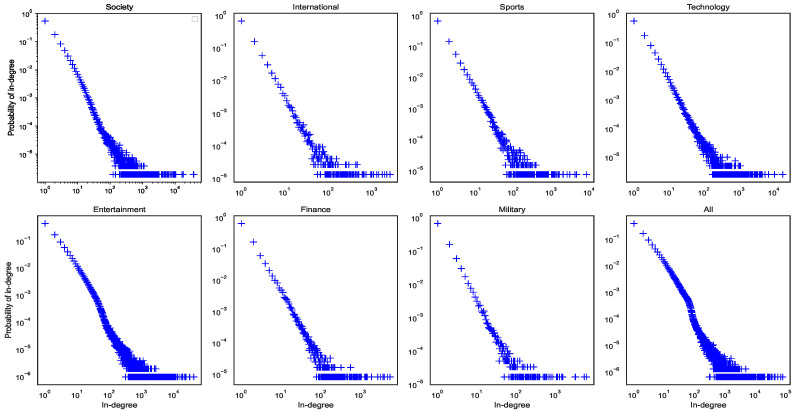
Probability distribution of in-degree of each domain-oriented retweet network. All refers to considering all domains as a whole.

The formation and development of elites is a dynamic process; this status is constantly changing by quantifying the interactive behaviors [41]. Many research methods for selecting elites are too simple and rely on official verification [7,11,12], and some users with “Big-V” may not be influential. User influence is essentially a reflection of interaction capabilities and therefore this paper targets the real elites through a lens of interactive networks. Weibo features a variety of interactive forms such as following, mentioning and retweeting. Needless to say, the frequency of being forwarded, through which tweets are disseminated in social media, is relatively more realistic and direct than the number of followers in reflecting user influence [63]. Moreover, the attributes in the Weibo data collected contain the retweeted status of original tweets and the corresponding author information; accordingly, a retweet network between users can be constructed by extracting their retweeting relationships. The retweet network can be represented by a directed weighted graph in which the nodes represent Weibo users (those without edges are omitted), the edges are the set of retweet relationships among users and the weight of the edge is the total number of occurrences of retweets between user pairs (in our sampling period). The larger the edge weight is, the more faithful the retweeter is to the original publisher. Accordingly, we built seven networks using the separate retweet data from the seven domains in our later explorations. Their degree distributions show the power–law trend with long tails (see Appendix A Figure A1), indicating the existence of a minority of elites with a large number of connections. Figure 1 shows a sampled snapshot of the military retweet network with an edge threshold larger than 10 retweets for better visualization. These constructed retweet networks provide decent preconditions for subsequent work, such as the selection of elites and the inference of the user influence indicators.

**Figure 2:**
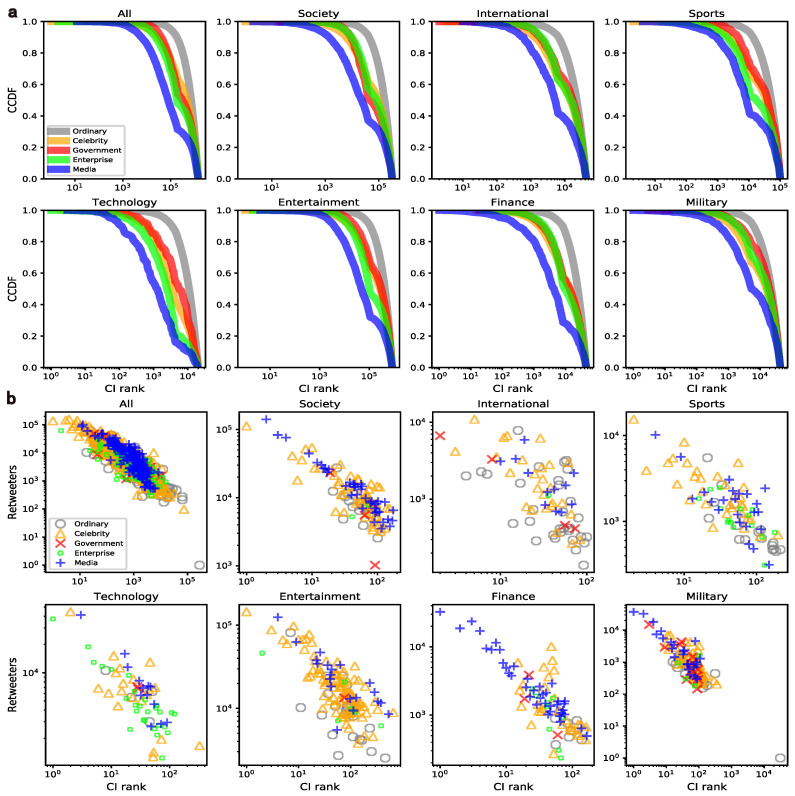
Mapping of user influence in retweet network and content domains. Note that, here, the CI is calculated within three hops as recommended and the sub-graphs (**a**,**b**) represent the mass level and the elite level, respectively.

**Figure A2:**
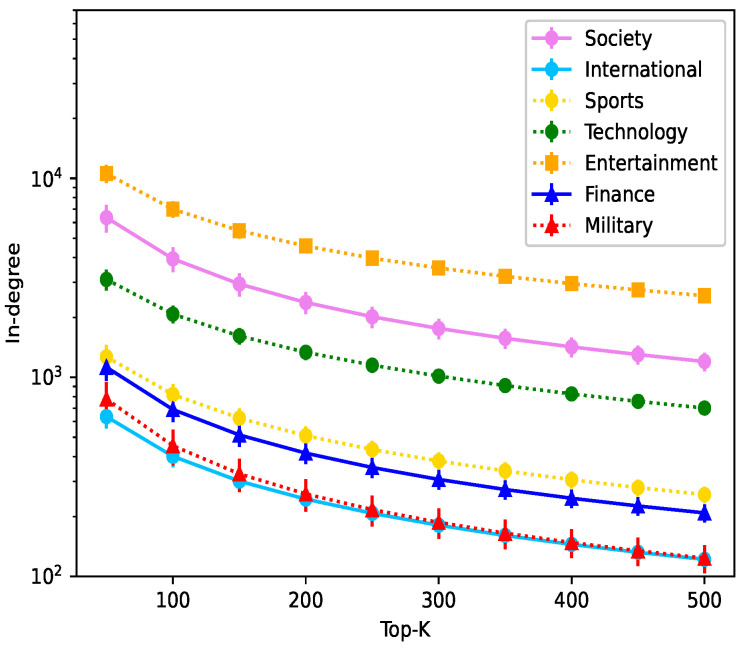
Influence scores (in-degree) of the top-k influentials in each domain-oriented retweet network.

**Figure A3:**
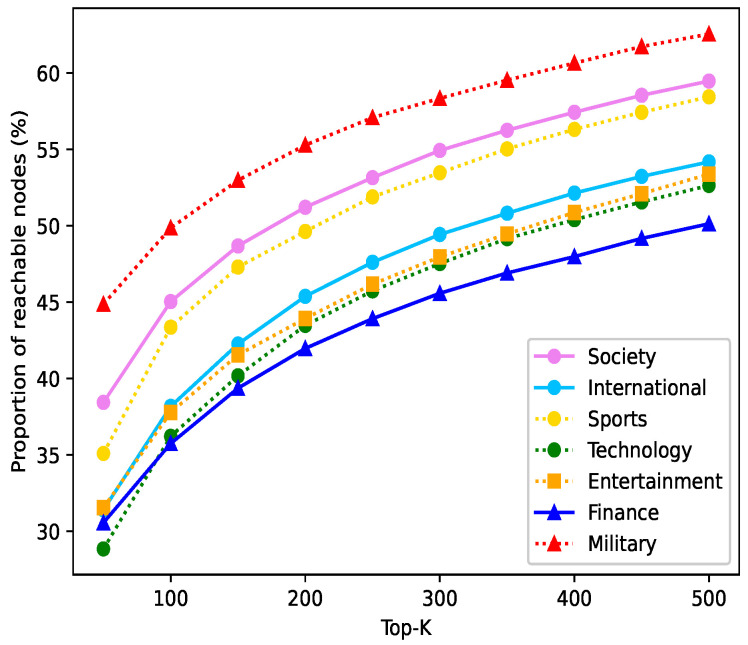
Proportion of users reached by the top-k influentials in each domain-oriented retweet network.

The key element in marketing and information diffusion is a minority of influentials [3]. After building a network through the retweet relationships between users, it is important to acknowledge that there can be many structural indicators for valuing user influence, such as in-degree, closeness, betweenness, many random walk methods and CI (Collective index) [21,30,64], and the centrality methods have high computational complexity [65]. For these seven large-scale networks in which the weight of each edge is more than 2, the CI and the in-degree are employed to rank users by influence in each domain, and their computational complexity is O(NlogN) and O(1) (where N is the number of users in the retweet network), respectively. In addition, the formula of CI is as

(2) $$CI_{l}\left(i\right)=\left(k_{i}-1\right)\sum_{j\in \partial B(i,l)}\left(k_{j}-1\right),$$

where $k_{i}$ is the degree of node *i* and ∂B(i,l) refers to the set of nodes in the ball of radius *l* centered on node *i*. Due to the uneven size of networks in various domains, we select the top k users as elites from all users. Moreover, Appendix A Figure A2 and Figure A3 show the changes in influence scores (in-degree) of top-k users and their proportion of reachable nodes, respectively. To ensure sufficient user influence and collective communication effects, k is set to 200. The CCDF (complementary cumulative distribution function) and scatter plots of the CI rankings for the mass and elites are demonstrated across domains in Figure 2. Note that the lower value of CI ranking represents more influence, and Figure 2b shows that both indicators are positively related, and elites selected from them are almost the same. According to in-degree, the distribution of elites in each group as k = 200 can be found in Table 1, and a total of 930 unique elites are obtained from all domains. In addition, the influence changes of elites are more diversified, which is significantly different from that of the mass. For instance, enterprises dominate in technology, and celebrities are even more influential than media users in sports and entertainment. These differences between the mass and elites imply that user groups and content domains should be comprehensively considered, and the following experiments on behavior variations will be profiled and demonstrated at both levels of elites and mass.

## 4. Behavior Variations between the Mass and Elites

After splitting the users into five groups and the contents into seven domains, how user behavior varies from the mass to elites can then be fully investigated. Focusing on the two primary behaviors of tweeting and retweeting, behavior variations will be specifically examined from the view of tweeting activity, homophily, loyalty and content characteristics, which together reconstruct a full angle of individual behavior on Weibo.

### 4.1. Tweeting

#### 4.1.1. Tweeting Activity

**Figure 3:**
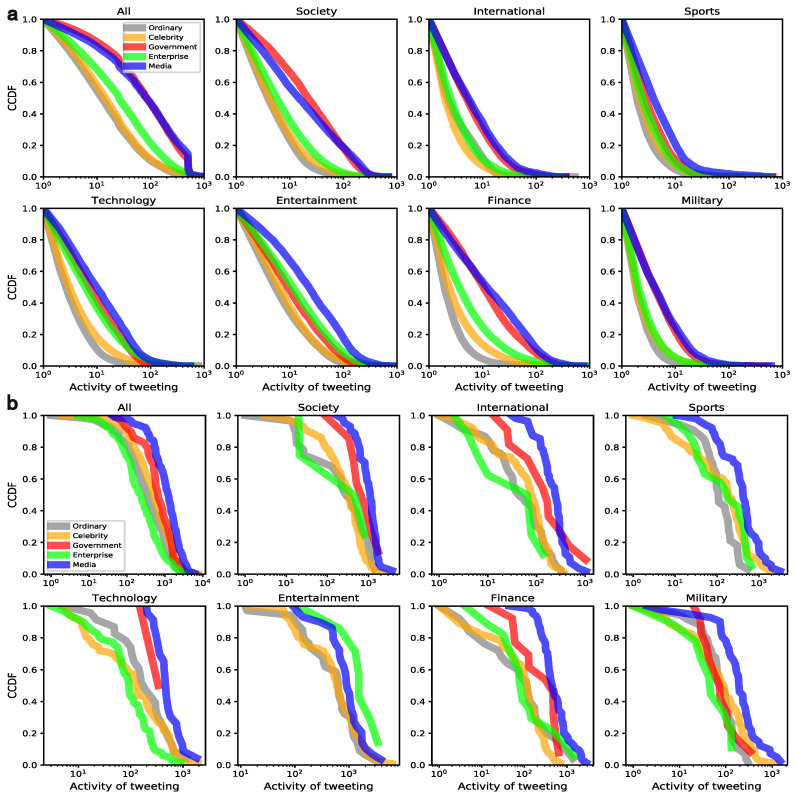
Mapping between tweeting activity and content domains. Note that sub-graphs (**a**,**b**) represent the mass level and the elite level, respectively.

Posting more tweets, i.e., being more active in social media, will bring more opportunities to be noticed [16,26,36]. Here, the activity of tweeting is simply measured by the number of tweets within the sampling period. For each user group, we obtain the CCDF of the activity in the seven domains at both the mass level and the elite level in Figure 3.

At the mass level, for all domains, as shown in Figure 3a, the media has the highest proportion of active users in almost every domain except society and finance. It is counterintuitive that the activity of celebrities is relatively low and even lower than that of the ordinary users in the international domain. However, at the level of elites, the activity of various users has different patterns across domains, which is different from the situation of the mass. Surprisingly, the government elites even vanish in the sports and entertainment domains. In general, the elites have a higher level of activity than the mass and their patterns of varying across domains are also different.

#### 4.1.2. Content Diversity

**Figure 4:**
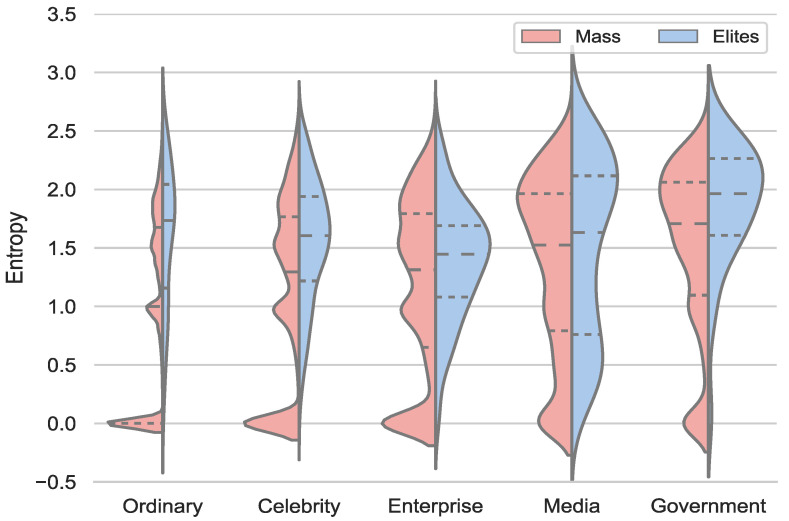
The distribution of posting entropy for the mass and elites across user groups and domains.

To measure the diversity of the content posted by various users, we calculate the posting entropy of different topics as

(3) $$H=-\sum_{i=1}^{6}p_{i}log\left(p_{i}\right),$$

where $p_{i}$ refers to the proportion of the posted tweets in domain *i*. The distribution of the posting entropy of various users is shown in Figure 4. To begin with, a certain percentage of mass users demonstrate a single interest, i.e., their posts are only related to one domain, and the posting entropy correspondingly equals 0. Contrarily, elites post content of richer topics than the mass, and their average value of entropy is accordingly higher than that of the mass. Except for the enterprise, the top quartile of elites in other verified types is larger than that of the mass, which is explained as the contents posted by elites in the group of enterprise are relatively unitary.

#### 4.1.3. Content Links

Users on Weibo would like to publish content containing short URLs (t.cn) jumping to images, news and videos to attract audiences [26]. To perform the analysis of content links, we transform the short links to the corresponding source URLs through the Python package urllib2. Due to the speed limit with regard to tracing the source addresses of short URLs on Weibo, in this study, 100,000 users at the mass level were randomly selected to compare with the elites.

**Figure 5:**
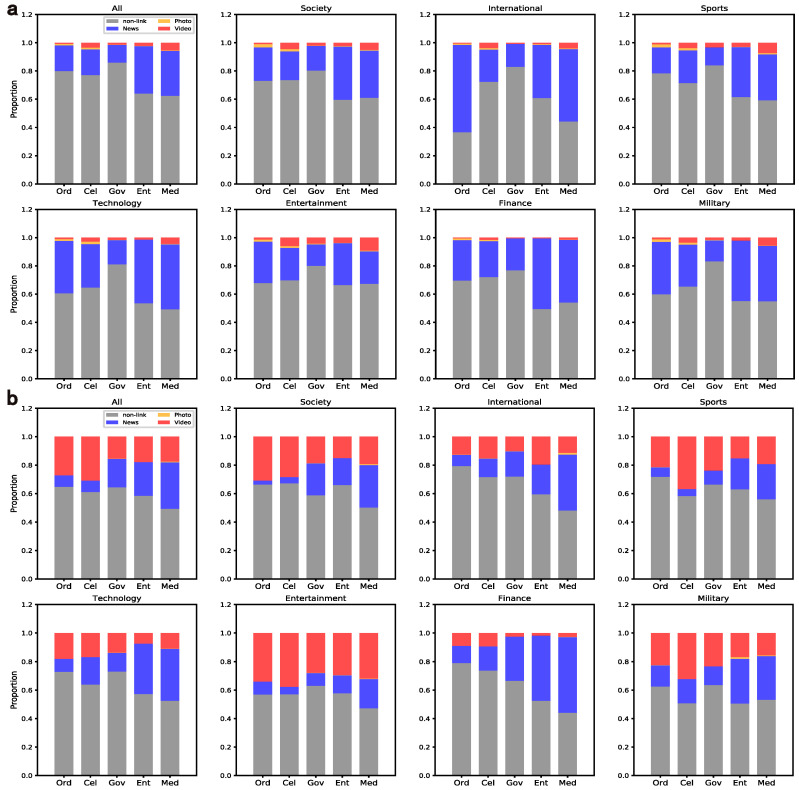
Percentages of tweets containing various links across user groups and domains. Note that sub-graphs (**a**,**b**) represent the mass level and the elite level, respectively.

The percentages of tweets containing links at the mass and elite levels are shown in Figure 5, which illustrates the differences in content links across domains and user groups. In general, the elites obviously prefer to post tweets with video links, especially the celebrities. In addition, the media has the largest proportion of using links no matter whether at the mass or elite level. On the contrary, the content of the government is more formal, usually only words, which is in line with the previous finding that many accounts just post government documents and may lose their audience [6]. These differences may result in different content popularities, and the correlations between these posting preferences and retweets obtained will be further examined in the actions of popularity promotion.

### 4.2. Retweeting

#### 4.2.1. Homophily

**Figure 6:**
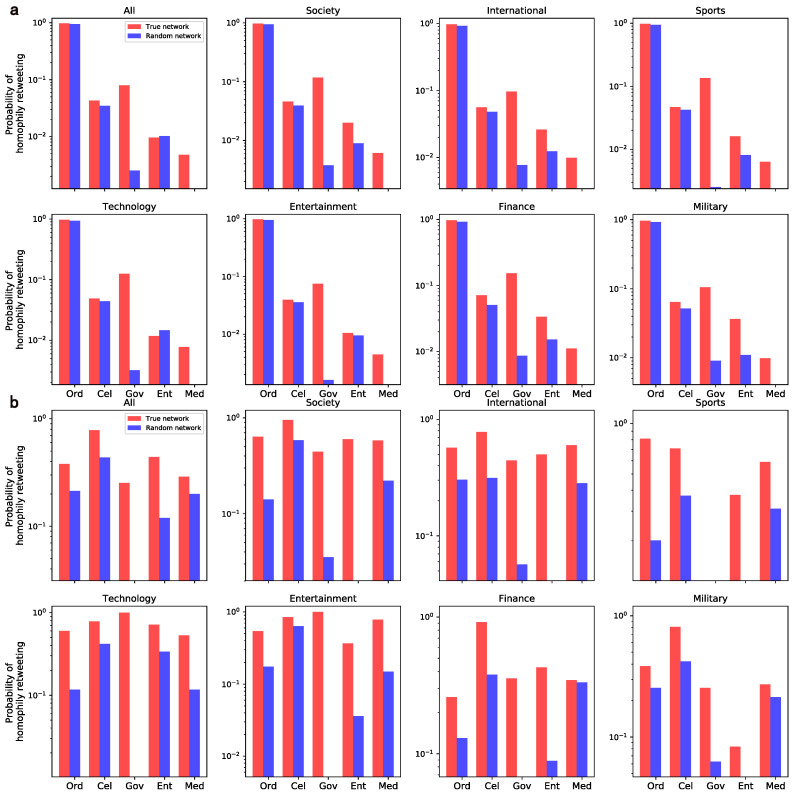
Homophily of retweeting inclination across user groups and domains. Note that sub-graphs (**a**,**b**) represent the mass level and the elite level, respectively.

On the basis of the constructed forwarding network, we measure the retweeting homophily through the probability of edges connecting a pair of users with the same verified type (regardless of the edge weights). At the same time, we also calculate this indicator in the random network in which all edges are randomly rewired as a benchmark to test its significance. The comparison of the homophily between the real network and its random counterpart in each domain is shown in Figure 6. The homophily of the government and media users is significantly higher than that in the random counterparts, indicating their inclination toward homogeneous retweeting. The group of ordinary users also possess a high homophily because they account for 95% of the nodes in the network, and accordingly, their random homophily is similarly high, meaning a low significance. The enterprise’s homophily is even lower than the random value in the technology domain. In fact, a large part of the corporate accounts come from the emerging Internet technologies and these accounts seldom interact with other enterprises due to their competitive relationship, unless they have an interest-based partnership. Nonetheless, the homophily of elites in enterprise is always higher than the corresponding random value, which indicates that the enterprise elites can interact freely without restrictions.

#### 4.2.2. Loyalty

The weight of the edge in the network refers to the retweeting frequency which indeed reflects the loyalty of the retweeter. Considering that the tweet count of the target user will affect weights, here we use the probability that each tweet of the target user will be forwarded by each retweeter to represent the loyalty.

**Figure 7:**
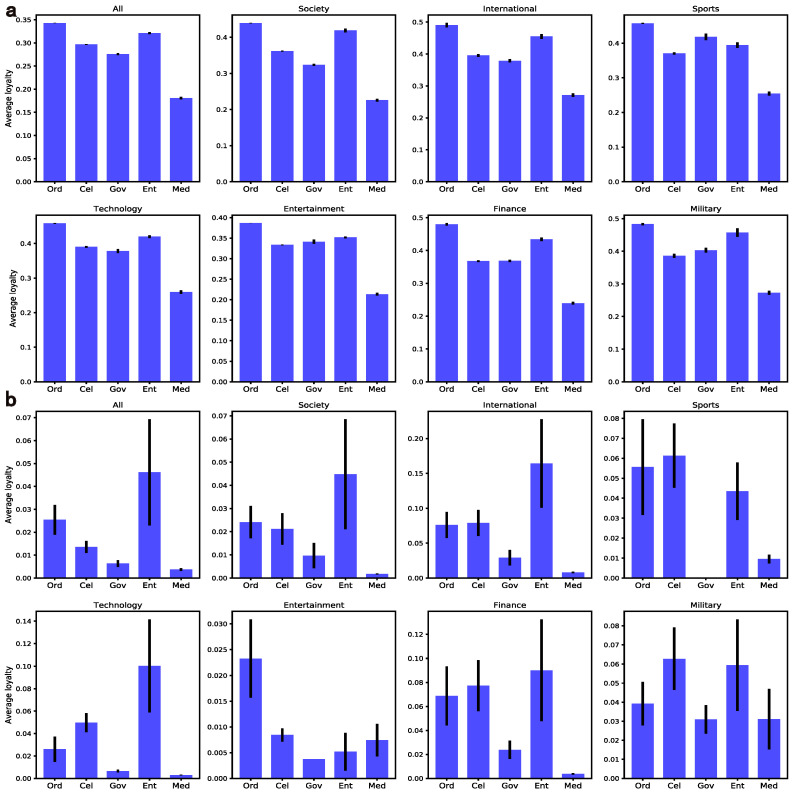
Average loyalty of retweeters across domains and user groups. Note that sub-graphs (**a**,**b**) represent the mass level and the elite level, respectively.

The average loyalty of all retweeters of various user groups is shown in Figure 7, where all target users have posted at least twice. It is clear that the mass has a higher average loyalty than elites, which suggests that the former are more intimate with retweeters. Interestingly, the loyalty value of the media is low, which can be explained by how the media users attract a large number of retweeters by being active, but their audiences are less sticky. Just as a passionate fan will share almost every tweet by a star, the accounts of branch companies will keep pace with the headquarters, especially at the elite level. More importantly, the loyalty fluctuates differently at two levels, inspiring the following explorations in user-oriented promotions. Concerning RQ1, systematic comparisons from the perspectives of tweeting activity, homophily, loyalty and content characteristics suggest that elites indeed behave differently from the mass on Weibo.

## 5. User-Oriented Actions for Popularity Promotions

After the comparison of various behaviors between the mass and elites, some key actions for promoting popularity inspired by the behavioral differences are presented and testified. The experiments focus on these following questions: For users at both the levels of elites and the mass, what kinds of content will obtain more retweets? How does one enrich the content? Which is more important for retweeters, the number or the loyalty?

### 5.1. Content Diversity

**Figure 8:**
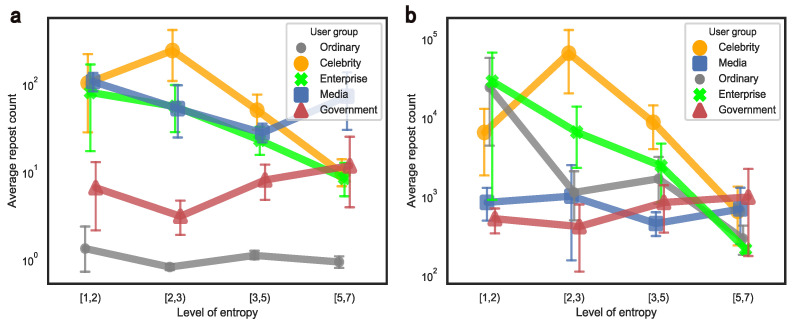
The relationship between the posting entropy and the average repost count across user groups. Note that sub-graphs (**a**,**b**) represent the mass level and the elite level, respectively.

In order to explore how content diversity affects retweets, we divide the individual posting entropy into several levels. Specifically, the entropy is 0 when the user only posts in one domain, 1 represents posting in two domains on average, 1.585 refers to three domains and so on. Considering that the richer the content is, the fewer the number of users there are, the grouping of users is divided into “[1, 2)”, “[2, 3)”, “[3, 5)” and “[5, 7]”, according to the corresponding entropy values of “[0, 1)”, “[1, 1.585)”, “[1.585, 2.32)” and “[2.32, 2.807]”. The average repost count and error bar of each group at the mass and elite levels are shown in Figure 8, respectively.

In general, the contents with rich domains do not directly lead to more retweets and even have a negative impact on the groups of the ordinary and enterprise. Enterprises are inherently professional and ordinary users who pay attention to too many areas will be distracted [56], which would reduce their content quality. However, the government will slightly increase the number of retweets if they post more diverse content, while celebrities who focus on two domains are ideal and will gain greater content popularity. Notably, the varying patterns of the media and ordinary users are different at the mass and elite levels. Therefore, each user group needs to pinpoint appropriate content domains and cannot pursue rich themes blindly.

### 5.2. Content Links

Upgrading or manipulating the formats of posted content to produce “vivid” stories is another feasible path for popularity promotion. Specifically, to enhance the popularity of posted content, actions such as adding the URLs of videos, news or pictures are pervasively adopted in social media [23,26]. However, as we have revealed that user behavior varies across groups and domains, these actions might lose their expected effect. With the help of behavior variations across groups and domains, how to select suitable actions to enhance the popularity of content will be illustrated in a user-oriented manner.

After merging all users’ tweets, the tweets of each user i can be represented by a vector $l_{i}=\left(l_{i}^{1},l_{i}^{2},l_{i}^{3},l_{i}^{4}\right)$, where $l_{i}^{1},l_{i}^{2},l_{i}^{3}$ and $l_{i}^{4}$ separately represent the fraction of tweets containing videos, news articles, pictures and non-links. For each tweet, how many times it was retweeted in our sampling period is the most convincing metric for valuing its popularity. Then, based on these preliminaries, to examine whether these enhancement actions work under circumstances of different user-domain assemblies, the pairwise Pearson correlation coefficients between $l_{i}$ and content popularity, i.e., the average repost count per tweet for user i, can be investigated at the mass and elite levels.

The results at the mass level are shown in Table 2. In neglecting content domains, the proportions containing videos are positively related to content popularity for all users except the media; this is especially the case for the government, implying a significant promotion from adding videos in tweets authored by government accounts. However, after assembling content domains and user groups into different circumstances, the effect of various actions fluctuates unexpectedly across domains. Interestingly, the enhancement effect on content popularity will be trivial in domains where users are active and will be relatively significant in domains where they are inactive. For example, adding the links of videos will lead to popularity promotion for ordinary users in the technology domain, for enterprise in the military domain and for celebrity in the international domain. Meanwhile, the lack of significant results in the finance domain also suggests the possibility that these strategies might completely lose their effect under certain circumstances. Unexpectedly, actions such as adding more links of news articles might even undermine content popularity for the ordinary and celebrity groups. This result implies the negative impact of unmatched actions in user-domain assemblies, suggesting again that behavior variations should be considered in promotion actions.

The correlations at the elite level are presented in Table 3. In ignoring domains, adding news articles helps only enterprise, and the video links of elites are not as effective as the mass, even if the former has a higher proportion of videos. However, across different user-domain assemblies, the effect of actions at the elite level demonstrates more interesting variations than those disclosed at the mass level. Specifically, on the one hand, in domains in which users are inactive, popularity will be similarly enhanced for elites. For example, adding video links will help government users earn a boost in popularity in the entertainment domain, and more links of news articles will help enterprises in the finance and military domains. On the other hand, for elites, the popularity of tweets in their active domains can also be further improved, which is inconsistent with the observations at the mass level. For example, adding the links to pictures can improve content popularity in the society domain for government users, enterprise can boost the popularity of technology-related tweets by adding links to news articles and the financial content of media users can be popularized by adding videos. Similarly, the lack of significant results for the sports and international domains again suggests that even at the elite level, the enhancement effect of these actions might be completely lost.

Different from the conclusion reached by Wang et al. [26] that tweets with picture links are more likely to be retweeted, the above results imply that these enhancement strategies actually have varying performances across user-domain assemblies. It is possible to lose the enhancement effect or to even cause a negative impact if unmatched strategies are inappropriately selected. From this illustration, the variation of user behavior across domains found in this study implies that it is necessary to update previous understandings of marketing in social media. In particular, the exact mapping between behavior variations and popularity promotions will offer prior knowledge to develop appropriate strategies from a more comprehensive perspective, one in which various assemblies of user groups and content domains can practically and systematically be considered.

### 5.3. Loyalty

**Figure 9:**
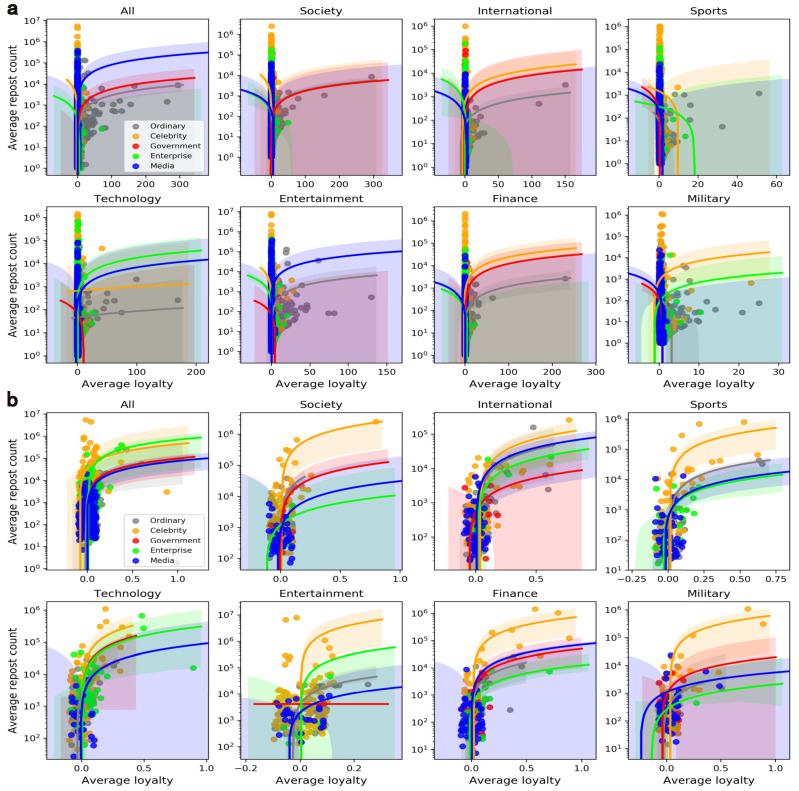
The relationship between the loyalty and the average repost count across user groups and domains. Note that sub-graphs (**a**,**b**) represent the mass level and the elite level, respectively.

For each user on Weibo, the averaged retweet count per tweet is another direct reflection of the content popularity. In order to explore the impact of loyalty on the popularity of target users, the regression curves of various users at two levels are shown in Figure 9. Compared with the mass, elites need more loyalty of retweeters to increase their content popularity, especially celebrities and corporates. However, the effects of loyalty on enterprise elites are unstable across domains. These patterns further indicate the heterogeneity of users even in the same verified group and suggest that the seeding of influentials and crowds is domain dependent.

Intuitively, the elites usually have a large number of retweeters which is also a key factor in popularity promotion, and this is why they were chosen. Therefore, we further explore how the retweeter count and loyalty influence the content popularity. Based on the retweet networks, a multiple regression analysis is performed with the averaged retweet count as the dependent variable, and the results of the mass and elites are shown in Table 4. From the perspective of loyalty, the coefficient of elites is higher than that of the mass, implying that the loyalty of a retweeter is important for elites to promote their content popularity. Moreover, the mass users should pursue more new retweeters. After all, their average loyalty is higher than elites. The results suggest that the strategies for promoting popularity of the mass and elites are significantly different from the behavior of retweeting loyalty. Regarding RQ2, our findings offer solid evidence for the differences between elites and the mass in actions of popularity promotion.

To address RQ3, we establish a complete picture of the different strategies and corresponding effects between the mass and elites across domains and groups from the perspectives of content diversity and links and loyalty, which can provide suitable ways for various users to increase their popularity.

## 6. Discussion

The behavioral comparison between influential elites and the mass grassroots is an important communication issue [7,8,10,11,12], and the popularity promotion is one of the primary goals in communication, especially in marketing scenarios [1,2,3,25]. However, the behavior variations of the mass and elites across user groups and domains and the relation between behavioral differences and strategies for enhancing popularity are rarely addressed. Meanwhile, the scarcity of massive behavioral data and expensive traditional methods make it difficult to study behavior variations in multiple domains. Fortunately, the network science and machine learning models help split 8,520,933 users into five groups, categorize over 140,000,000 tweets into seven domains, target elites with real influence and offer ideal circumstances for investigating the comprehensive mapping between behavior variations and popularity promotions. To the best of our knowledge, a complete picture of behavior variations across user groups and domains at the mass and elite levels is first established. Additionally, how diverse behaviors influence the actions for popularity promotions is thoroughly examined and testified, which can be applicable to both influentials and crowd targeting from the perspective of marketing practitioners.

Regarding the theoretical implications, our study enriches the literature on the behavior analysis of the mass and elites in multiple domains. Although existing studies have described the great difference in role and influence between the mass and the elites in information diffusion [3,7,11,12], little attention has been paid to the changes across content domains and user groups. In this paper, we document direct evidence that there are significant differences between the mass and elites in various behavioral dimensions across user groups and domains. Specifically, we find that media users are mostly active, and the varying patterns of the mass and elites are quite different across domains. As for the entropy of tweeting, most elites have a wider variety of content than the mass, and they often use video links to tell stories vividly. In addition, only the homophily of enterprises is very low. Surprisingly, the average loyalty of the mass is higher than that of elites. This study extends the dimensions of the behavior comparison between the mass and the elites, contributing to the existing research on mass communication. In addition, to the best of our knowledge, this study takes the first step to explore the differences in popularity strategies between the mass and elites, which creates opportunities for marketers to design different strategies for users with different behavioral characteristics.

One important practical implication of our findings is that the mass and elites need to adopt their own appropriate strategies to promote popularity. Our study further provides empirical evidence of the differences in promotion strategies across user groups and domains. Unexpectedly, rich content with domain diversity would not always bring more retweets, which is even counterproductive for enterprises and ordinary users. This result is consistent with the previous study which suggests that people prefer the more professional websites for online shopping [66]. Moreover, correlation analysis of various links and retweets displays different communication effects across user-domain assemblies, which is not in line with the views that the picture link can increase the possibility of being retweeted [26]. The promotion effect of video on the mass is stronger than that of elites who have a higher proportion of video links. Interestingly, commonly employed actions might also work well in domains in which users are inactive, which implies that shortcomings in activity can, to some extent, be fixed by content manipulations. For instance, government elites may gain significant popularity improvements by embedding the links of videos in their inactive domains such as entertainment and sports. Finally, we suggest that elites need to improve the quality of their fans and the mass should foster and reach new audiences to promote their popularity. The other practical implication is that our framework provides methodological support for future research on behavioral differences in multiple roles and domains and even domain-based target marketing. Because it considers all types of users and content, the mapping obtained in this study is fine-grained and directly application-oriented. More importantly, by investigating the mapping at the levels of both masses and elites, different varying patterns of behavior across domains are also revealed. These patterns indicate the heterogeneity of users even in the same verified group and suggest that the seeding of influentials and crowds is domain-dependent. Based on an illustration of how to select appropriate strategies for boosting content popularity, it is ensured that our findings will inherently offer insights for marketing-like scenarios on social media.

## 7. Conclusions

In summary, users in social media need to find an individualized enhancement strategy that fits their behavioral characteristics rather than a mere copycat. Our findings fill the knowledge gaps of how the behavioral differences between the elite and the mass influence their marketing strategies in multiple domains and offer guidelines on both targeting seeds and strengthening promotions in realistic marketing-like scenarios.

This paper has made a preliminary study on the relation between behavioral variations and popularity promotions, and a few limitations should be considered in reviewing the results. For example, the topic classification of this paper is suitable for the discourse system of Chinese Weibo, and the findings might not be directly extendable to other platforms and countries. Meanwhile, the mapping discussed here is assumed to be static, so a promising direction for future research is to gain an in-depth understanding of its spatiotemporal dynamics. Moreover, finding more strategies to enhance content popularity and analyzing them simultaneously is one of the future goals.

## Figures and Tables

**Table 1:** Summary statistics of user groups.

User Group	Ordinary	Celebrity	Government	Enterprise	Media
Mass	8,043,807	301,118	20,370	87,155	9983
Elite	196	408	29	111	186

**Table 2:** Pearson correlation coefficients between actions and popularity at the mass level.

Content Domains	All Users	Video	News Article	Picture
All	Ordinary	0.013 ***	−0.006	0.002
	Celebrity	0.050 ***	−0.024	−0.006
	Government	0.224 **	−0.043	0.114
	Enterprise	0.096 **	−0.043	0.038
	Media	0.072	−0.065	0.018
Society	Ordinary	0.003	−0.006	0
	Celebrity	0.123 ***	−0.025	−0.005
	Government	0.071	−0.059	0
	Enterprise	−0.01	0.071	0
	Media	−0.038	0.073	0
International	Ordinary	0.006	0	0
	Celebrity	0.080 **	0.015	−0.005
	Government	0.025	0.054	0.056
	Enterprise	−0.012	0.084	−0.013
	Media	0.164	−0.073	−0.003
Sports	Ordinary	0.041 ***	−0.019 ***	0.001
	Celebrity	0.04	−0.021	−0.005
	Government	0.062	0.036	0
	Enterprise	0.089	−0.061	0.004
	Media	−0.046	−0.059	0.489 ***
Technology	Ordinary	0.017 ***	−0.020 ***	0.004
	Celebrity	0.002	−0.024	−0.003
	Government	0.149	0.002	0.08
	Enterprise	0.017	−0.039	−0.005
	Media	−0.011	0.02	0.01
Entertainment	Ordinary	0.010 **	−0.009 *	0.003
	Celebrity	0.03	−0.038 *	−0.008
	Government	0.057	−0.062	−0.025
	Enterprise	−0.001	−0.04	−0.003
	Media	0.05	−0.061	0.008
Finance	Ordinary	0.005	−0.006	0
	Celebrity	0.028	−0.022	−0.004
	Government	0.01	−0.08	0.034
	Enterprise	−0.008	−0.012	−0.005
	Media	−0.051	−0.028	−0.052
Military	Ordinary	0.005	−0.019 **	0.003
	Celebrity	0.006	−0.02	−0.008
	Government	0.145	−0.018	−0.04
	Enterprise	0.316 ***	0.008	0
	Media	−0.043	−0.12	−0.024

Note: User groups and content domains are assembled to simulate various circumstances. Significance levels are two-tailed; * *p* < 0.05, ** *p* < 0.01 and *** *p* < 0.001.

**Table 3:** Pearson correlation coefficients between actions and popularity at the elite level.

Content Domains	Elites	Video	News Article	Picture
All	Ordinary	−0.075	−0.081	−0.019
	Celebrity	−0.073	0.004	−0.023
	Government	−0.003	0.16	0.272
	Enterprise	−0.142	0.240 *	−0.032
	Media	0.124	−0.085	−0.042
Society	Ordinary	−0.058	−0.049	−0.012
	Celebrity	−0.094	0.096	−0.008
	Government	−0.166	0.21	0.674 ***
	Enterprise	0.188	−0.003	−0.028
	Media	0.078	−0.096	−0.032
International	Ordinary	−0.014	−0.042	−0.02
	Celebrity	−0.061	−0.055	−0.008
	Government	0.263	−0.197	0.013
	Enterprise	0.034	−0.061	0
	Media	0.137	−0.058	−0.043
Sports	Ordinary	−0.139	−0.082	−0.033
	Celebrity	−0.072	−0.041	−0.012
	Government	0.103	−0.02	0
	Enterprise	−0.088	0.182	−0.001
	Media	0.041	−0.025	−0.037
Technology	Ordinary	−0.1	−0.148 *	−0.015
	Celebrity	−0.031	0.018	−0.016
	Government	0.048	0.147	−0.113
	Enterprise	−0.089	0.302 **	−0.022
	Media	0.150 *	−0.074	−0.029
Entertainment	Ordinary	−0.048	−0.189 **	−0.028
	Celebrity	−0.065	0.005	−0.013
	Government	0.502 **	−0.024	0.006
	Enterprise	−0.105	−0.028	−0.028
	Media	0.07	−0.087	−0.033
Finance	Ordinary	−0.045	−0.055	−0.012
	Celebrity	−0.031	−0.058	−0.007
	Government	−0.057	−0.075	−0.09
	Enterprise	0.125	0.358 **	0
	Media	0.165 *	0.019	−0.007
Military	Ordinary	0.095	−0.069	−0.041
	Celebrity	−0.071	−0.045	−0.011
	Government	−0.056	−0.031	0.334
	Enterprise	−0.077	0.324 **	−0.025
	Media	0.057	0	−0.018

Note: User groups and content domains are assembled to simulate various circumstances. Significance levels are two-tailed; * *p* < 0.05, ** *p* < 0.01 and *** *p* < 0.001.

**Table 4:** Results of multiple regression analysis at the mass and elite levels.

	Mass				Elite			
	**Coef**	**Std Err**	t	***p*** > $\left|t\right|$	**Coef**	**Std Err**	t	***p*** > $\left|t\right|$
const	1.2787	0.007	193.957	***	22.8254	10.615	2.15	(0.032) *
average loyalty	0.8224	0.012	66.248	***	1629.9279	90.067	18.097	***
retweeter count	0.0037	0	340.457	***	0.0018	0	4.291	***
observations	3,024,960				928			
$R^{2}$	0.038				0.267			
adjust $R^{2}$	0.038				0.266			
F-statistic	59,660				168.6			

Note: Significance levels are two-tailed; * *p* < 0.05 and *** *p* < 0.001.

## Data Availability

The data sets used in this study are publicly available after careful anonymization and can be downloaded freely through https://doi.org/10.6084/m9.figshare.12155349.v1, accessed on 4 May 2020. A previous version of the submission can also be found in the arXiv preprint through https://arxiv.org/abs/2004.05591, accessed on 4 May 2020.

## Appendix A

Appendix tables and figures to this article are available in the appendix.

**Table A1:** The precision, recall and F-measure of the cross-validation. All refers to considering all domains as a whole.

Domain	Count	Precision (%)	Recall (%)	F-Measure (%)
Society	22,975	65.31	74.71	69.69
Finance	66,134	87.04	86.77	86.90
Military	34,617	90.04	92.43	91.22
Entertainment	91,679	88.53	95.33	91.80
International	14,253	65.83	59.00	62.23
Sports	108,041	98.62	93.90	96.20
Technology	73,674	92.36	86.83	89.51
All	411,373	83.96	84.14	84.05
